# A Rare Case of an Iliac Fracture at the Iliac Fossa Immediately after Salter Innominate Osteotomy

**DOI:** 10.1155/2021/6653726

**Published:** 2021-01-27

**Authors:** Masanori Wako, Kensuke Koyama, Taro Fujimaki, Naoto Furuya, Hirotaka Haro

**Affiliations:** Department of Orthopaedic Surgery, Faculty of Medicine, University of Yamanashi, 1110 Shimokato, Chuo-shi, Yamanashi 409-3898, Japan

## Abstract

This report presents the unusual case of a 5-year-old girl with iliac fracture just after Salter innominate osteotomy for developmental dysplasia of the hip. The iliac fracture was diagnosed two days after Salter innominate osteotomy, and computed tomography (CT) revealed that it was at the extremely thin portion of the iliac wing called the “iliac fossa.” We were able to reduce the fracture by pulling the left leg distally, and after reducing the iliac bone, the ilium was fixed by Kirschner wire from the anteroinferior iliac spine and anterosuperior iliac spine. The patient was in a hip-spica cast for 6 weeks postoperatively and allowed to walk from 3 months after the surgery. At the last follow-up one year after the surgery, bone union was completely obtained, and she had no complications. The cause of the fracture seems to be the stress concentration on the iliac fossa due to the cranked iliac osteotomy line passing through the iliac fossa. The current case indicates the importance of careful evaluation by CT before surgery and ensuring that the osteotomy line does not extend near the iliac fossa.

## 1. Introduction

Salter innominate osteotomy was introduced by Salter as a treatment for developmental dysplasia of the hip (DDH) [1], and it has been a common surgical procedure for DDH or Legg-Calve-Perthes disease (LCPD). As a complication of the procedure, some troubles with fixating wire or the lateral femoral cutaneous nerve have been reported [2–6]. However, there have been no reports on iliac fracture just after Salter innominate osteotomy. Here, we report an unusual case of a 5-year-old girl with an iliac fracture just after Salter innominate osteotomy.

## 2. Report of the Case

The mother of the patient was fully informed that the patient's data would be submitted for publication, and she provided her consent.

**Figure 1:**
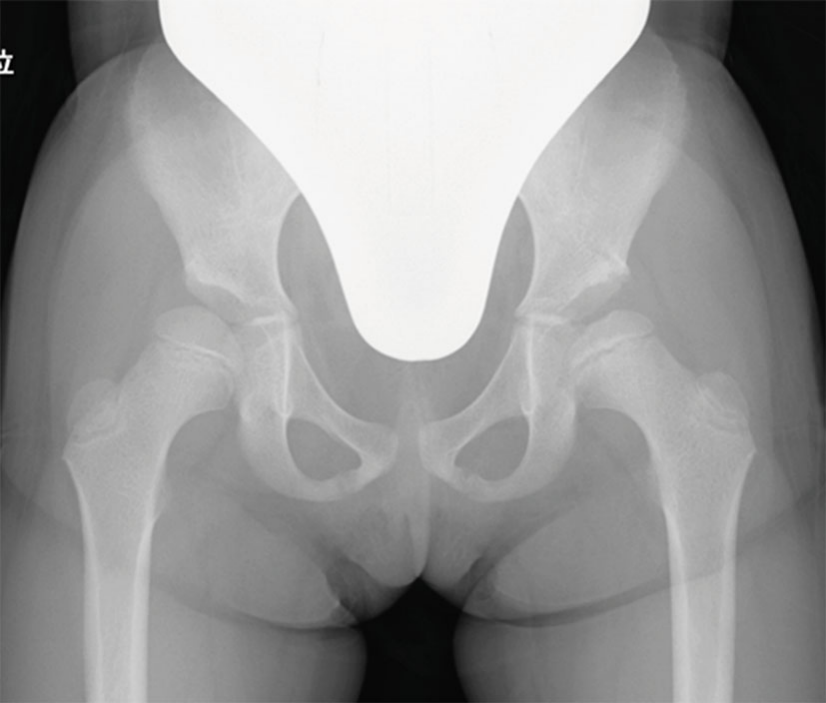
Preoperative radiograph of bilateral hips.

A girl with a familial medical history of DDH from her mother was delivered by Cesarean section weighing 2110 g. She was diagnosed with left DDH three months after birth, and her left hip was successfully reduced by a Pavlik harness. She grew up without any motor or neurological problems, but acetabular dysplasia remained in her bilateral hips (Figure 1). The right and left acetabular indices were 32° and 37°, respectively, and the center edge angle was 0° and -3° when she was five years and 1 month old. Hence, we planned a Salter innominate osteotomy for her bilateral hips.

In our institution, to evaluate the anterior or posterior coverage of the acetabulum, we routinely perform CT examination before surgery for all patients undergoing Salter innominate osteotomy. We routinely perform iliac osteotomy in the crank shape after the iliac is exposed supraperiosteally. And we carefully pulled the distal bone fragment manually by adducting and rotating externally the hip and fix the iliac bone with three or four 2.4 mm Kirschner wires. Autogenous or artificial bone grafting was not performed. A hip-spica cast was applied 4 weeks after the surgery.

**Figure 2:**
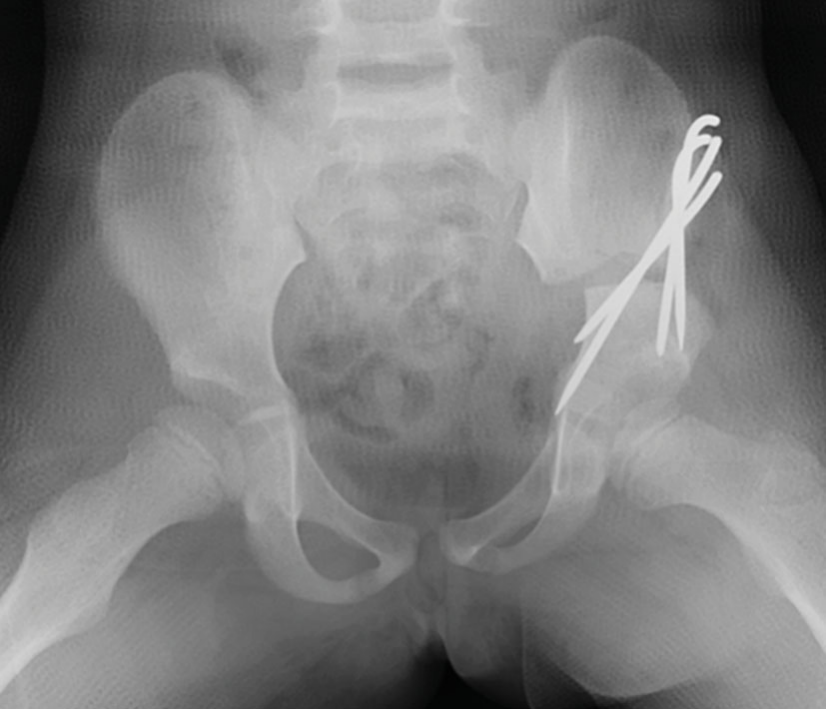
Radiograph of the hips just after Salter innominate osteotomy.

**Figure 3:**
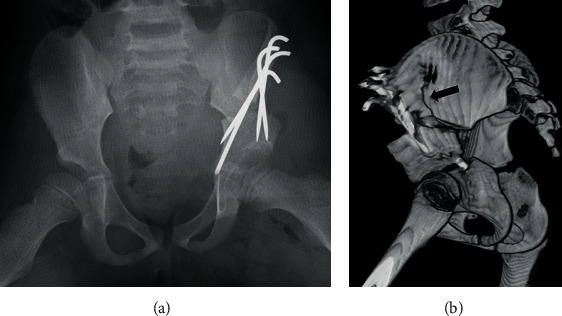
(a) The radiograph at two days after Salter innominate osteotomy. (b) 3D CT image indicating iliac fracture between the osteotomy site and iliac crest (black arrow).

**Figure 4:**
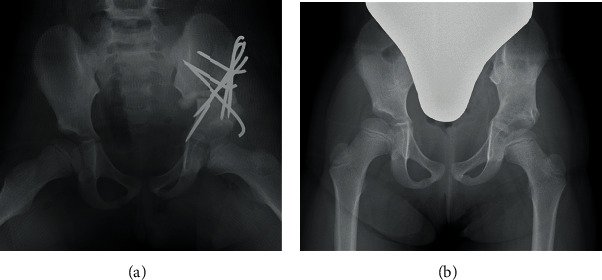
(a) The radiograph just after the additional surgery. (b) The radiograph of the hips at the last follow-up.

First, we performed Salter innominate osteotomy for the left hip without any intraoperative complications (Figure 2). Two days after the surgery, she had unusual severe pain; therefore, we performed a radiographic examination and found a left iliac fracture (Figure 3(a)). Computed tomography (CT) revealed an iliac fracture between the osteotomy site and the iliac crest (Figure 3(b)), and we performed emergency surgery on the same day. During the surgery, we confirmed that the fracture occurred in a very thin portion of the iliac bone. The osteotomy site was still very stable, and there was no loosening of the Kirschner wires. We were able to reduce the fracture by pulling the left leg distally, and after reducing the iliac bone, one 2.0 mm Kirschner wire from the anteroinferior iliac spine (AIIS) and two 2.0 mm Kirschner wires from the anterosuperior iliac spine (ASIS) were inserted beyond the fracture line (Figure 4(a)). Following the additional surgery, the hip-spica cast was removed after 6 weeks, and she was permitted to walk after 10 weeks. All Kirschner wires were removed 6 months after the initial surgery, and at the last follow-up one year after the surgery, bone union was completely obtained, and she had no complications (Figure 4(b)). We are wondering about the next surgery on the opposite hip in the future.

## 3. Discussion

Salter innominate osteotomy is a common surgical procedure for DDH or LCPD. Although its long-term result is stable, there are some reports on the complications of the surgery. Wire deviation, displacement of the grafted bone, loss of position of the osteotomy site, and dysesthesias in the distribution of the lateral femoral cutaneous nerve have been reported [2–6]. Böhm et al. reported that 2 of the 73 hips in their series had wire migration, and 2 hips had a displacement of the bone graft [6]. Pekmezci and Yazici reported that the complications related to pin placement may reach 7.4%, while the incidence of graft displacement ranges between 0 and 19% [5].

However, to the best of our knowledge, there has been no report on iliac fracture around the osteotomy site. In the current case, the fracture was found 2 days after the surgery and was located between the top of the crank shape osteotomy line and the iliac crest behind the wire insert portion.

**Figure 5:**
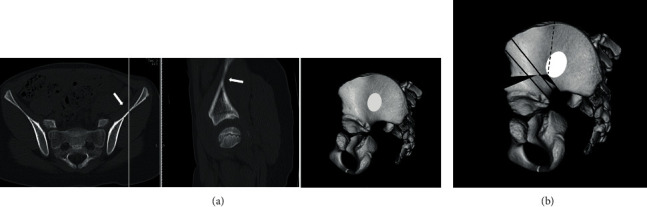
(a) Preoperative CT image of the pelvis of the patient. The white arrows and white ovoid area indicate the thin iliac portion (iliac fossa). (b) The schema of the pelvis of the current case after Salter innominate osteotomy. The black dot line indicates the fracture line, and black lines show the Kirschner wires.

**Figure 6:**
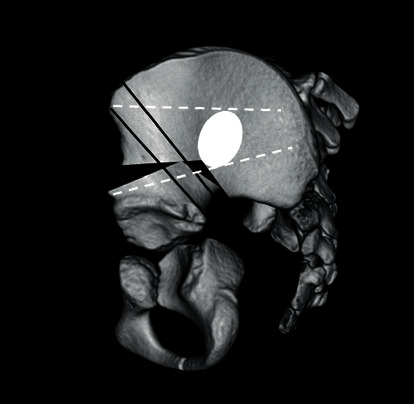
White dot lines indicate the Kirschner wire inserted after the pelvic fracture. We think wire insertion at this position can also prevent fracture like the current case.

Reviewing the preoperative CT image again, the current case had an iliac bone which was partially very thin. Bircher expresses the extremely thin portion of the iliac wing as the “iliac fossa” and suggests that pin insertion into the iliac bone should be avoided in this area in the treatment of pelvic fracture [7]. The crank shape osteotomy of the current case was applied near the iliac fossa, and the force of the distal bone fragment to move to the original position may concentrate on the iliac fossa from the top of the crank shape osteotomy line. We speculate that the force is a cause of the fracture (Figure 5(a)). Moreover, we think that too rigid fixation with thicker Kirschner wire with less flexibility is also considered to be one of the causes of the fracture because the force of returning the bone fragments concentrated on the thin part of the iliac bone. In the current case, the fracture was successfully fixated by inserting a Kirschner wire from the AIIS and ASIS in the direction, which is the route generally used to insert the half pin of the external fixator for pelvic fracture [7–9] (Figure 5(b)). This wire insertion technique is considered to be useful not only as a treatment procedure after the iliac fracture but also as a preventive procedure for iliac fractures when performing Salter innominate osteotomy for a patient with a very thin iliac bone (Figure 6).

The current case indicates that the following three points should be considered when performing Salter innominate osteotomy in order to reduce the risk of iliac fracture. (1) Pelvic morphology should be carefully evaluated by CT before surgery. (2) Care should be taken that the osteotomy line does not extend near the thin part of the iliac bone. If the “iliac fossa” can be identified with preoperative CT, I recommend straight osteotomy. (3) If the iliac is very thin, additional wire fixation from the AIIS or ASIS to the proximal iliac should be considered as a useful optional method to prevent similar fractures.
